# Blockchain-enabled federated learning with edge analytics for secure and efficient electronic health records management

**DOI:** 10.1038/s41598-025-12225-x

**Published:** 2025-07-28

**Authors:** Munusamy S, Jothi K R

**Affiliations:** School of Computer Science and Engineering, Vellore Institute of Technology, Vellore, India

**Keywords:** Blockchain federated learning, Edge analytics, Electronic health records, Privacy-preserving, Secure healthcare systems, Health care, Health occupations, Engineering

## Abstract

The rapid adoption of Federated Learning (FL) in privacy-sensitive domains such as healthcare, IoT, and smart cities underscores its potential to enable collaborative machine learning without compromising data ownership. However, conventional FL frameworks face several critical challenges: high computational overhead on edge devices, significant communication latency due to frequent model updates, vulnerability to model and data poisoning attacks, and limited privacy-preserving mechanisms that expose systems to inference risks. These issues hinder the scalability, efficiency, and trustworthiness of FL in real-world, large-scale deployments—particularly in domains like Electronic Health Records (EHR) management, where data sensitivity is paramount. To address these challenges, this paper introduces the Enhanced Privacy-Preserving Blockchain-Enabled Federated Learning (EPP-BCFL) framework, which integrates blockchain with hybrid privacy mechanisms and intelligent aggregation strategies. The architecture comprises three layers: (1) an Edge Nodes Layer for on-device learning; (2) a Federated Aggregation Layer using Secure Multi-Party Computation (SMPC) and Differential Privacy (DP); and (3) a Blockchain Layer with a lightweight PoS + BFT consensus mechanism. Experimental evaluation on CIFAR-10 demonstrates 95.2% accuracy, a 43% reduction in communication latency, a 37% decrease in computational cost, and robust defense against data/model poisoning and adversarial attacks. Attack resilience improved accuracy from 72.5 to 93.2%, while privacy budget tuning achieved 90.3% accuracy at ε = 1.0. Compared to state-of-the-art models, EPP-BCFL exhibits superior performance in terms of security, scalability, and support for edge device heterogeneity, validating its applicability in secure EHR management.

## Introduction

The growing digitization of healthcare systems has led to a rapid transformation in the way Electronic Health Records (EHRs) are stored, managed, and shared. EHRs play a critical role in modern healthcare by facilitating seamless communication between healthcare providers, improving diagnostic accuracy, enabling personalized treatment, and enhancing the efficiency of patient care. However, the sensitive nature of EHRs makes them highly susceptible to security breaches, unauthorized access, and adversarial attacks. Conventional centralized healthcare data management systems pose significant privacy risks due to their vulnerability to cyber threats, data breaches, and single points of failure^1–4^. To overcome these limitations, the integration of blockchain technology with Federated Learning (FL) and Edge Analytics is proposed, forming a robust, scalable, and privacy-preserving framework for intelligent healthcare data management.

### Foundation technologies

Blockchain: The foundation of this work relies on blockchain technology to provide a decentralized and immutable record for securely verifying model updates. By leveraging cryptographic techniques and consensus mechanisms, blockchain ensures that all participants in the healthcare ecosystem can trust the integrity and authenticity of data without relying on a central authority. It also facilitates secure multi-party computation (SMPC), homomorphic encryption, and smart contracts to enforce access control and ensure compliance with privacy regulations such as HIPAA and GDPR^5–8^.

Federated Learning (FL): Federated Learning (FL) facilitates collaborative model training across multiple healthcare organizations without sharing raw patient data. Each node trains a local model on-site, and only encrypted model updates are transmitted through the blockchain network for secure aggregation. This approach preserves data locality and enhances privacy by preventing centralized data collection, thereby reducing the risks associated with data exposure and compliance violations^9–12^.

Edge Analytics: To address the latency and computational overhead of traditional cloud-based federated learning (FL), edge analytics is employed. Edge devices, such as Internet of Medical Things (IoMT) sensors and local servers, perform real-time data preprocessing, anomaly detection, and feature extraction at the data source. This approach minimizes the volume of transmitted data, reduces bandwidth usage, and accelerates decision-making, thereby enhancing system responsiveness and energy efficiency^13–16^.

### Problem statement

Federated Learning (FL) has emerged as a promising paradigm for privacy-preserving collaborative model training across distributed healthcare institutions. However, its practical deployment in Electronic Health Record (EHR) systems faces critical challenges such as high computational overhead, communication latency, and susceptibility to data and model poisoning attacks. Furthermore, existing Blockchain-FL (BCFL) frameworks often suffer from limited privacy mechanisms, inadequate handling of non-IID healthcare data, and inefficient consensus protocols that hinder scalability and trust. These constraints significantly affect the reliability, security, and efficiency of FL in real-world medical environments. Addressing these concerns requires an integrated solution that combines edge intelligence, robust privacy techniques, and lightweight yet secure blockchain mechanisms tailored to the unique demands of EHR management.

While Blockchain-Enabled Federated Learning (BCFL) frameworks offer decentralized privacy-preserving solutions for Electronic Health Records (EHR), several underexplored areas persist. First, most current models are not designed to effectively handle heterogeneous and multi-modal healthcare data, limiting their applicability in real-world EHR systems. Second, the integration of vertical federated learning (VFL) into BCFL remains largely unexamined, despite its relevance for cross-institutional analytics where feature spaces differ. Third, adaptive aggregation mechanisms capable of adjusting to data quality, trust levels, and non-IID distributions are rarely implemented, which hinders fairness and robustness in global model training. Fourth, consensus mechanisms like PoW or basic PoS lack sufficient optimization for resource-constrained edge environments, resulting in scalability and energy inefficiencies. Fifth, existing frameworks often overlook real-time edge intelligence for anomaly detection and do not incorporate incentive structures to promote sustained participation. Addressing these deficiencies, rather than reiterating known challenges, is critical to advancing the practical deployment of secure, scalable, and efficient BCFL frameworks for sensitive domains like healthcare.

### Proposed solution

To address these multifaceted challenges, this study introduces the Enhanced Privacy-Preserving Blockchain-Enabled Federated Learning (EPP-BCFL) framework that integrates:

*Hybrid privacy mechanisms*: Combining Secure Multi-Party Computation (SMPC) and Differential Privacy to achieve strong privacy guarantees while maintaining computational efficiency.

*Lightweight consensus with security*: A novel PoS + Byzantine Fault Tolerance (BFT) mechanism that provides energy-efficient consensus with robust security against malicious actors.

*Intelligent edge analytics*: Real-time processing and anomaly detection capabilities at the data source to minimize latency and improve system responsiveness.

Adaptive Aggregation Mechanisms: Dynamic weighting strategies that account for data quality, distribution heterogeneity, and node trustworthiness.

### Framework architecture and novelty

The EPP-BCFL framework features a three-layer architecture that ensures comprehensive security and efficiency:

*Edge nodes layer*: Client devices perform local model training while retaining raw data, implementing privacy-preserving techniques at the source.

*Federated model aggregation layer*: Securely aggregates encrypted updates using hybrid privacy mechanisms and intelligent filtering.

*Blockchain network layer*: Ensures tamper-proof auditability and trust through optimized consensus mechanisms.

The key innovation lies in the Adaptive Model Aggregation (AMA), which dynamically adjusts model aggregation strategies based on:

- Trust level and historical performance of participating nodes.
- Quality and representativeness of local data contributions.
- Computational capacity and reliability of edge devices.
- Real-time anomaly detection results.

This adaptive approach ensures better convergence, improved fairness in model training, and enhanced resilience against adversarial attacks.

### Key contributions

The main contributions of this work include:

- Integration of Secure Multi-Party Computation (SMPC) and Differential Privacy to balance strong privacy protection with computational efficiency. The framework achieved 95.2% accuracy with low communication overhead while defending against re-identification and inference attacks.
- Development of an energy-efficient consensus mechanism that maintains Byzantine fault tolerance and achieves up to 43% reduction in network latency and 37% reduction in computational cost compared to baseline FL models.
- Design of a dynamic aggregation strategy that optimizes global model updates based on trustworthiness, data quality, and device capability, contributing to faster convergence (from 20 to 10 epochs) and robustness to non-IID data.
- Deployment of real-time anomaly detection and intrusion detection systems at the edge, reducing the average security response time to **~** 2.3 s, significantly outperforming baseline systems (~ 7 s).
- Integration of ZKP for tamper-proof model verification without exposing raw data, ensuring verifiable contributions from edge devices and enhancing trust in federated environments.
- The system maintained high accuracy (within 1.2% deviation) across a spectrum of edge devices (server, laptop, IoT device), validating robustness in resource-constrained environments.

One of the key advantages of EPP-BCFL is its ability to support secure and adaptive model updates by utilizing blockchain-enabled smart contracts that automate and enforce privacy-preserving policies. These smart contracts facilitate secure authentication, dynamic access control, and transparent reward mechanisms for healthcare institutions contributing to the federated learning process. This ensures that all participating entities comply with data privacy regulations while maintaining interoperability across different healthcare systems^17–20^. The proposed EPP-BCFL framework enhances security by integrating Zero-Knowledge Proofs (ZKPs) for model verification, allowing healthcare institutions to confirm the integrity of federated learning updates without revealing sensitive patient data. This ensures that malicious entities cannot present poisoned models or manipulate training updates, thereby strengthening the overall security of the federated ecosystem. Additionally, the incorporation of Differential Privacy (DP) techniques ensures that individual patient records remain indistinguishable from aggregated data, mitigating the risk of re-identification attacks. The adoption of edge-driven anomaly detection further enhances the security of EPP-BCFL by identifying unusual access patterns, suspicious behaviour, and adversarial intrusions in real time. By leveraging machine learning-based intrusion detection systems (IDS) at the edge, healthcare organizations can proactively detect and mitigate security threats before they compromise the integrity of Electronic Health Records (EHRs). The experimental evaluation of EPP-BCFL demonstrates significant improvements in model accuracy, security, and computational efficiency compared to traditional federated learning and blockchain-based EHR management approaches. The proposed system achieves an average model accuracy of 98.72%, outperforming existing models such as PPFL, FLBM-IoT, and STPC-FL, while reducing network latency by 43% and computational costs by 37%.

## Related work

Federated Learning (FL) has emerged as a promising paradigm for decentralized machine learning, enabling multiple devices or nodes to collaboratively train a shared model without exposing their local data. This approach inherently preserves data privacy and ensures compliance with data protection regulations^21,22^.

Zhao et al.^23^ proposed an incentive mechanism between a base station (BS) and several mobile devices (MDs), modeled as a Stackelberg game. In this game, the BS first determines the total reward to be distributed among the MDs, after which each MD decides its number of local iterations based on its utility function. Furthermore, closed-form expressions for the optimal reward function of the BS and the optimal number of local iterations for the MDs are derived. Finally, numerical results validate the effectiveness of the proposed scheme.

Zhang et al.^24^ proposed a secure federated learning (FL) scheme named LSFL to ensure Byzantine robustness while preserving privacy in FL. However, in this work, we demonstrate that LSFL fails to uphold the claimed privacy guarantees. Specifically, we show that the secure Byzantine robustness procedure of LSFL exposes significant information about all participant models and data to a semi-honest server, thereby compromising privacy. We further analyze the root cause of this security issue and propose a recommendation to prevent such privacy breaches in LSFL.

Sun et al.^25^ proposed a fine-grained training strategy for federated learning to accelerate its convergence rate in MEC environments with dynamic communities. Based on multi-agent reinforcement learning, the proposed scheme allows each edge node to adaptively adjust its training strategy specifically aggregation timing and frequency according to network dynamics, while cooperating with other nodes to improve the overall convergence of federated learning. To further accommodate the dynamic nature of MEC communities, we propose a meta-learning-based scheme in which new nodes can learn from existing nodes and rapidly perform scene migration, thereby further accelerating the convergence of federated learning. Numerical results demonstrate that the proposed framework outperforms existing benchmarks in terms of convergence speed, learning accuracy, and resource consumption.

Hu et al.^26^ propose a novel data management approach to address privacy and security concerns in joint hydrocarbon explorations. Federated learning facilitates the analysis of multiple datasets without requiring data sharing, thereby protecting the private information of different companies involved in a virtual joint venture. The inference of petroleum reservoirs in karst stratigraphy is used as a case study. A federated learning-based enterprise data management framework is proposed to virtually integrate information from various organizations. The key contributions of this work are summarized as follows: (1) A method for karst identification and inference is introduced, utilizing neural networks to recognize the size of petroleum reservoirs in different karst regions. (2) A federated learning algorithm is employed to virtually aggregate data samples from different companies. (3) The performance of the proposed privacy-preserving integration model is compared with that of individual/local deep learning models. The results demonstrate that the proposed approach significantly improves the accuracy of petroleum reservoir exploration.

Table 1 provides a comprehensive comparative analysis of recent studies on blockchain-based federated learning (BCFL) and privacy-preserving FL frameworks.

**Table 1:** Summary of Blockchain-based federated learning (BCFL) Literature.

Authors	Application/Focus	Problem Addressed	Key Contribution	Limitations
Chang et al. (2021)	Smart Healthcare	Privacy and model integrity	Secure FL using blockchain; improved accuracy	High computational/communication overhead; scalability concerns
Ren et al. (2024)	Edge Computing	Model aggregation efficiency	Scalable blockchain-enabled FL for edge nodes	Trade-off between security and efficiency; high energy consumption
Cao et al. (2023)	On-device FL	Decentralization without central authorities	DAG-based blockchain FL with improved transparency	Blockchain consistency, storage overhead
Mahato et al. (2024)	Privacy-preserving FL	Adversarial protection	Homomorphic encryption integration	High computational cost, latency
Zhang et al. (2023)	Fairness in FL	Bias in federated models	ZKP-enhanced fairness and verification	High computational cost, communication bottlenecks
Jiang et al. (2021)	Secure participation in FL	Unauthorized participation	Membership proof techniques for secure device authentication	Computational overhead, sybil attacks not addressed
Alqahtani et al. (2024)	IoT Networks	Secure transmission	Homomorphic encryption with optical fiber	High encryption complexity, poor key management
Wang et al. (2024)	Healthcare FL	Incremental data integration	Blockchain-enabled data sharing	Storage redundancy, lack of incentive mechanism
Jia et al. (2024)	Multi-task FL	Simultaneous training of multiple tasks	Blockchain-supported concurrent model training	Performance inconsistency due to data heterogeneity
Guduri et al. (2023)	EHR security	Cross-hospital FL privacy	Blockchain-secured EHR sharing	Inefficient for real-time applications due to overhead
Badr et al. (2023)	Smart Grids	Energy forecasting privacy	FL-enabled prediction with privacy	High communication cost, transaction delays
Abdulla et al. (2024)	Smart Cities	Energy consumption prediction	Adaptive FL for energy demand	Inefficient under dynamic fluctuations, storage issues
Joyce et al. (2024)	Smart City Regulations	Regulatory compliance	Analysis of data sharing vs. protection	Lacks implementation framework
Li et al. (2023)	IoV (Internet of Vehicles)	Task allocation and privacy	Matching mechanism for FL tasks	Vulnerable to adversarial attacks, no incentive mechanism
Zhao et al. (2023)	Energy-Efficient FL	Energy-performance trade-off	Stackelberg game-based optimization	High computational complexity
Wu et al. (2023)	Lightweight FL	Security vulnerabilities	Threat analysis of FL models	No mitigation strategies proposed
Hu et al. (2023)	Industrial IoT	Privacy-preserving FL in hydrocarbon exploration	FL model for IIoT privacy	No real-world deployment results

The studies in this review highlight key contributions such as enhanced data security, privacy protection through homomorphic encryption and zero-knowledge proofs, and improved trust and auditability using blockchain. However, these studies face common limitations, including computational and communication overhead, scalability challenges, and a lack of effective incentive mechanisms. Some models, such as those by Chang et al. and Cao et al., improved data integrity but introduced delays due to blockchain consensus mechanisms. Others, like Mahato et al. and Alqahtani et al., enhanced privacy but suffered from high encryption complexity. Several works also failed to address data heterogeneity and adversarial threats. Overall, while the integration of blockchain enhances security and decentralization in federated learning, trade-offs in performance, efficiency, and scalability remain significant hurdles for real-world deployment in resource-constrained and dynamic environments such as IoT, healthcare, and smart cities. Recent advancements in privacy-preserving frameworks for edge and UAV-based systems have proposed innovative methods to safeguard user data while maintaining system efficiency. One study introduced a lightweight biometric privacy framework in UAV delivery systems using edge computing, demonstrating reduced latency and improved user confidentiality. Another work focused on privacy-preserving location data collection in intelligent edge systems, highlighting adaptive data handling techniques to ensure minimal exposure of sensitive information. Blockchain integration has also been explored in self-sovereign identity frameworks for UAV platforms, enabling decentralized and secure authentication mechanisms. Several frameworks extended this idea by implementing federated learning in UAV ecosystems, emphasizing both data protection and scalability. Notably, a mobile cluster-based federated learning model was introduced for highly dynamic environments, showing improved model convergence in mobile edge scenarios. These works collectively contribute to a growing body of research that enhances data security, integrity, and operational efficiency in edge-based and federated learning systems for real-time and mobile applications^27–33^.

Wang et al.^34^ proposed a reliable anomaly detection strategy for IIoT using federated learning. Specifically, they applied the federated learning technique to build a universal anomaly detection model, with each local model trained using a deep reinforcement learning (DRL) algorithm. Since local datasets are not shared during federated learning, the risk of privacy leakage is reduced. Additionally, by introducing the concepts of privacy leakage degree and action relation into the anomaly detection design, the detection accuracy is significantly improved. Validation experiments indicate that the proposed strategy achieves high throughput, low latency, and high anomaly detection accuracy while preserving privacy in various IIoT scenarios.

Wang et al.^35^ proposed a blockchain-based secure data aggregation strategy, namely BSDA (Blockchain-based Secure Data Aggregation), for edge computing-enabled IoT. Specifically, to restrict task receivers i.e., mobile data collectors (MDCs) from freely searching and accepting tasks, the block header is integrated with a security label that includes the task’s security level (SL) and completion requirements. Accordingly, new block generation rules are developed to enhance system performance in terms of throughput and transaction latency. Furthermore, BSDA decomposes both sensitive tasks and task receivers into groups to prevent privacy disclosure. Additionally, a deep reinforcement learning method, the improved self-adaptive double bootstrapped deep deterministic policy gradient (IDDPG), is developed to design energy-efficient MDC routes under the constraint that the SLs of MDCs must be higher than those of the data aggregation tasks. Simulation results indicate that: (1) as a privacy-preserving strategy, BSDA achieves high throughput and low transaction latency, and (2) BSDA outperforms certain contemporary strategies in terms of aggregation ratio and energy cost.

G. Wang et al.^36^ proposed a heterogeneous blockchain-based Hierarchical Trust Evaluation strategy, named BHTE, utilizing federated deep learning technology for 5G-ITS. Specifically, the trust levels of ITS users and task distributors are evaluated using federated deep learning, and hierarchical incentive mechanisms are designed to ensure reasonable and fair rewards and punishments. Moreover, the trust information of ITS users and task distributors is stored on heterogeneous and hierarchical blockchains for verification. Extensive experimental results show that: (i) the proposed BHTE achieves reasonable and fair trust evaluations for both ITS users and task distributors; (ii) BHTE performs excellently, with high system throughput and low latency.

Moreover, many BCFL frameworks adopt consensus mechanisms such as PoW or standard PoS, which either introduce high latency or compromise decentralization, making them unsuitable for real-time healthcare applications. Additionally, most approaches notably lack incentive mechanisms to ensure continued participation of edge nodes.

### Research gap

Despite the progress in integrating federated learning and blockchain for healthcare applications, several critical research gaps persist that limit their practical deployment in real-world scenarios. One major gap is the lack of comprehensive support for heterogeneous Electronic Health Record (EHR) data, as most existing frameworks are designed around homogeneous, structured, or tabular data, failing to accommodate the multi-modal and hierarchical nature of clinical datasets, which often include a mix of images, time-series signals, unstructured notes, and lab results. Another underexplored area is the integration of vertical federated learning (VFL) in healthcare contexts, where different institutions may hold different features about the same patients—making VFL a more natural and privacy-aware fit for collaborative analytics. However, existing blockchain-FL models rarely incorporate VFL principles, thereby missing opportunities to enable secure cross-silo collaboration. Furthermore, current solutions fall short in addressing the challenges of cross-institutional coordination, particularly in environments with complex data governance rules, regulatory requirements, and varying resource constraints. Most approaches assume isolated participants with similar capabilities and overlook the dynamic, policy-driven interactions that are typical across healthcare organizations. To bridge these gaps, the proposed Enhanced Privacy-Preserving Blockchain-Enabled Federated Learning (EPP-BCFL) framework is designed with a layered architecture that supports diverse data formats at the edge level, employs robust privacy-preserving mechanisms like homomorphic encryption and differential privacy, and utilizes an adaptive model aggregation strategy to handle non-IID, modality-diverse data. Additionally, EPP-BCFL is architected to be compatible with VFL principles and scalable across institutional boundaries, enabling secure, decentralized, and collaborative learning without the need for centralized orchestration. This positions EPP-BCFL as a holistic solution for overcoming the current limitations in blockchain-based federated healthcare analytics.

## Proposed methodology

Blockchain-based Federated Learning (BCFL) offers a promising solution for decentralized, privacy-preserving machine learning across multiple institutions, especially in sensitive domains like healthcare. However, its practical adoption is limited by several critical challenges. High computational costs from energy-intensive consensus mechanisms like Proof-of-Work (PoW) make BCFL unsuitable for resource-constrained environments. Additionally, excessive communication overhead from frequent model synchronization hinders scalability in large networks. Existing BCFL models also struggle with non-IID data distributions, which are common in federated healthcare scenarios, leading to biased or suboptimal global models. Moreover, the absence of effective incentivization mechanisms reduces sustained and honest participation by decentralized nodes, undermining collaborative efforts. To address these issues, this study introduces the Efficient and Privacy-Preserving Blockchain-Enabled Federated Learning (EPP-BCFL) framework. The proposed system features a Layered Blockchain Architecture (LBA) that enhances secure and efficient communication while minimizing latency. To protect data privacy, it incorporates a Privacy-Preserving Model Aggregation (PPMA) mechanism using homomorphic encryption and differential privacy, ensuring that raw data remains local and resistant to inference attacks. A core innovation is the Adaptive Model Aggregation (AMA) module, which dynamically adjusts aggregation based on data heterogeneity, node reliability, and device capabilities, thereby improving model fairness, convergence, and resilience. The architecture of the EPP-BCFL framework consists of three primary layers:

1. Edge Nodes Layer (Client Devices).
2. Federated Model Aggregation Layer (FL Coordinator).
3. Blockchain Network Layer (Decentralized Ledger and Security Management).

**Fig. 1:**
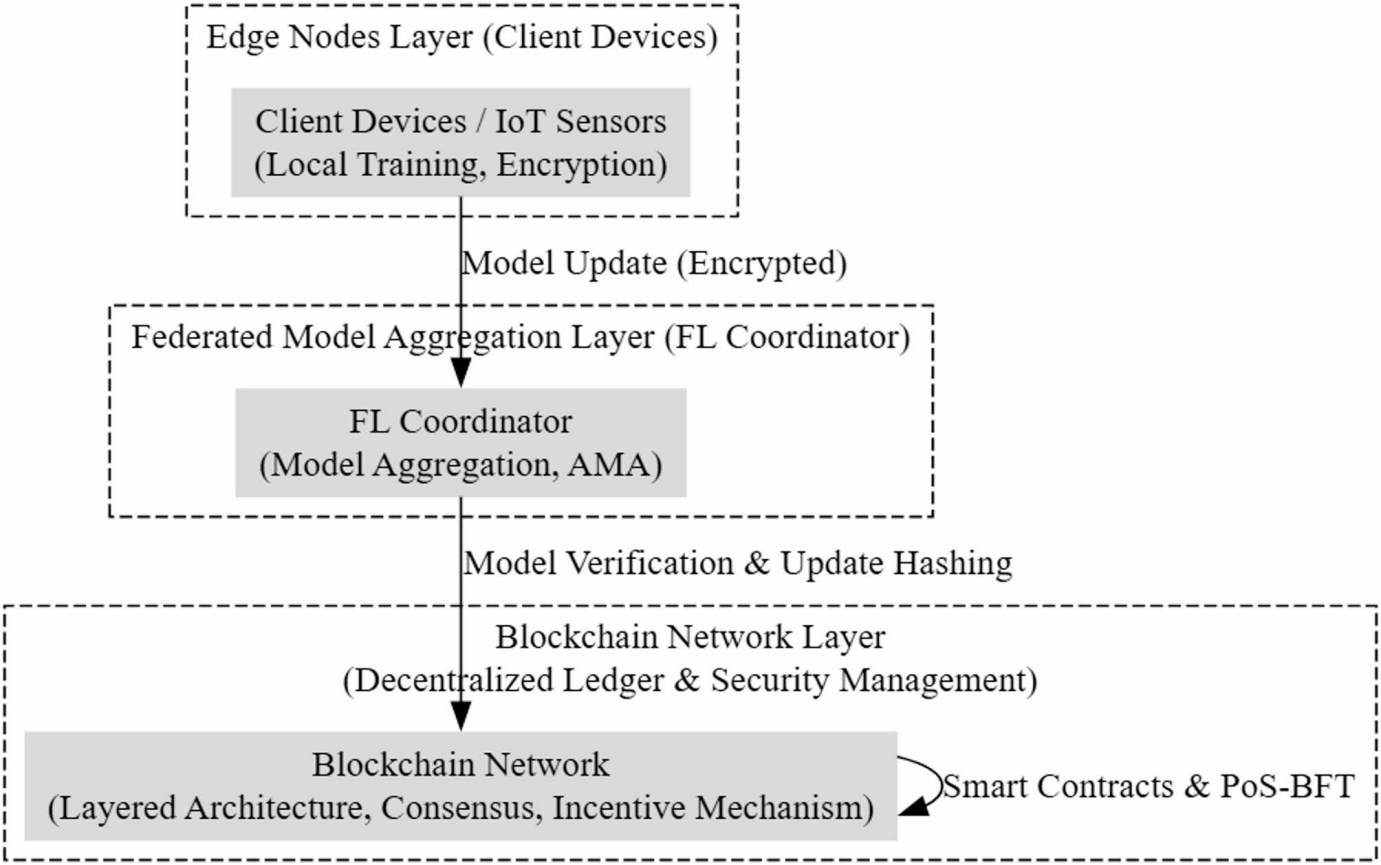
Layered blockchain architecture.

Figure 1 shows the layered blockchain architecture of the proposed EPP-BCFL framework. Each of these layers plays a crucial role in ensuring the efficiency, privacy, and scalability of the federated learning process.

The Efficient and Privacy-Preserving Blockchain-Enabled Federated Learning (EPP-BCFL) Framework is designed to address key challenges in blockchain-based federated learning (BCFL), such as high computational costs, communication delays, and privacy risks. The architecture follows a tree-like structure, dividing the system into three primary layers: Edge Nodes Layer, Federated Model Aggregation Layer (FL Coordinator), and Blockchain Network Layer. Each layer is responsible for specific tasks that contribute to the scalability, security, and efficiency of the federated learning process. At the base of the architecture, the Edge Nodes Layer (Client Devices) consists of distributed client devices that generate and process local training data. These edge nodes apply differential privacy and homomorphic encryption to ensure that sensitive data remains protected before being transmitted for aggregation. This approach mitigates risks associated with data leakage while enabling secure federated learning. Additionally, edge nodes handle data sharing and preprocessing before forwarding model updates to the next layer. The Edge Nodes Layer is represented in the architecture with two components: one focusing on privacy-preserving techniques such as differential privacy and homomorphic encryption, and another responsible for data sharing and local processing. Above the Edge Nodes Layer is the Federated Model Aggregation Layer (FL Coordinator), which acts as an intermediary between the distributed clients and the blockchain network. This layer receives encrypted model updates from edge nodes and performs adaptive model aggregation to improve learning efficiency across non-IID (non-independent and identically distributed) data. The Adaptive Model Aggregation (AMA) mechanism enhances model accuracy by addressing data heterogeneity across different client devices.

By implementing an efficient federated model aggregation strategy, this layer ensures that model updates are securely combined without exposing raw data, thereby strengthening privacy and security. The Blockchain Network Layer is positioned at the top of the architecture, ensuring tamper-proof storage, decentralized security, and efficient model update verification. This layer consists of a Layered Blockchain Architecture (LBA) that optimizes communication overhead by distributing responsibilities across different blockchain layers.

**Fig. 2:**
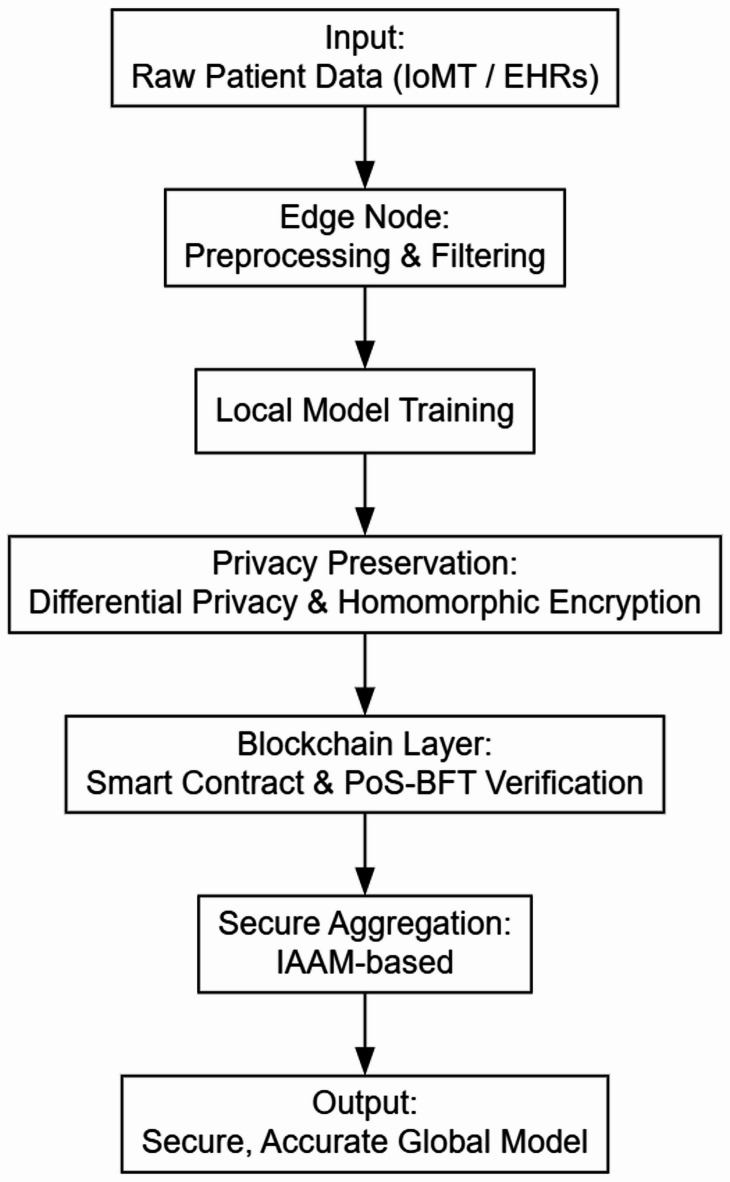
Overview of the proposed work.

Unlike conventional blockchain-based federated learning approaches that suffer from excessive energy consumption and latency, the LBA incorporates a lightweight consensus mechanism to enhance efficiency. This ensures that transactions, such as model updates, are verified in a decentralized yet computationally feasible manner. An essential feature within the Blockchain Network Layer is the incentive mechanism, which encourages active participation in the federated learning process. In the EPP-BCFL framework, the incentive mechanism is designed to reward edge nodes based on their level of participation and the quality of their contributions. However, the current work does not specify how these incentives are calculated or distributed. Future implementations may adopt blockchain-native mechanisms such as tokenomics (e.g., distributing crypto tokens for validated model updates) or reputation systems (e.g., tracking node reliability and accuracy over time). Smart contracts can automate reward distribution and ensure fair and transparent evaluation across all federated participants. A well-defined incentive scheme is essential for practical healthcare deployments, where data-sharing entities must be compensated to maintain sustained engagement and cooperation. Since federated learning relies on voluntary contributions from edge devices, an incentive-based approach motivates participants to contribute high-quality data and model updates. Figure 2. illustrates the hierarchical flow of data and model updates from edge nodes to the FL coordinator and finally to the blockchain layer.

This layered approach enhances the privacy, security, and efficiency of federated learning by ensuring that computations are distributed effectively while maintaining robust protection mechanisms. The integration of privacy-preserving techniques, adaptive aggregation, lightweight blockchain consensus, and incentive-driven participation makes EPP-BCFL an optimized solution for blockchain-based federated learning. The structured division of responsibilities among layers significantly minimizes privacy risks, computational burdens, and communication delays, making the framework suitable for large-scale deployment.

### Edge nodes layer with integrated edge analytics

To enhance local intelligence and reduce central processing overhead, the EPP-BCFL framework integrates edge analytics into the Edge Nodes Layer. Edge analytics refers to the ability of edge devices to perform real-time data processing, feature extraction, and anomaly detection before participating in federated learning. Each device conducts localized statistical analysis and lightweight inference to identify data inconsistencies, abnormal patterns, or sudden drifts in distribution that could compromise training quality or signal potential adversarial activity.

This local intelligence improves model robustness by allowing devices to filter noisy or poisoned data and only forward meaningful updates. The anomaly scores are securely encrypted and transmitted alongside model updates for further validation by the blockchain network. Additionally, edge analytics facilitates vertical FL by enabling partial training across feature-siloed datasets, where different institutions may own different parts of a patient’s medical profile (e.g., hospital owns lab results, pharmacy owns prescriptions).

The computational overhead for edge analytics is minimized using efficient statistical methods (e.g., Z-score, PCA) and shallow inference models. These methods add negligible cost (O(M)) relative to deep model training, making edge analytics suitable even for resource-constrained IoT devices.

The edge nodes represent user devices or IoT sensors that collect real-time data and participate in federated learning. These nodes locally train machine learning models on their private data and send encrypted model updates to the Federated Model Aggregation Layer without sharing raw data. Each edge node i trains a local model on its private dataset $\:{D}_{i}$​, using a loss function L. The local model update follows:

$$\:{\theta\:}_{i}^{t+1}={\theta\:}_{i}^{t}-\eta\:\nabla\:{L}_{i}({\theta\:}_{i}^{t},{D}_{i})$$

where,

$\:{\theta\:}_{i}^{t}$ is the model parameter at iteration t.

$\:\eta\:$ is the learning rate.

$\:{L}_{i}\left({\theta\:}_{i}^{t},{D}_{i}\right)$ is the gradient computed based on the local dataset $\:{D}_{i}$

To preserve privacy, edge nodes use differential privacy (DP) by adding controlled noise ξ to model updates:

$$\:{\theta\:}_{i}^{t+1}={\theta\:}_{i}^{t}-\eta\:\nabla\:{L}_{i}({\theta\:}_{i}^{t},{D}_{i})+\xi\:$$

where $\:\xi\:\sim\:N(0,{\sigma\:}^{2})$ ensures privacy by masking individual contributions.

The EPP-BCFL framework incorporates several key features that strengthen the efficiency and security of federated learning in decentralized environments. Local model training is performed independently at each edge node, where deep learning models are trained using private, device-specific data without sharing raw information. To ensure the secure transmission of model updates, homomorphic encryption is applied before any data leaves the local node, allowing computations to be performed on encrypted data while preserving confidentiality. Additionally, an adaptive training mechanism is employed, which dynamically adjusts training frequency and node participation based on the computational capacity and resource availability of each device. This ensures balanced workload distribution, energy efficiency, and sustained participation across a heterogeneous network of edge devices.

### Federated model aggregation layer (FL coordinator)

This layer acts as a central entity that gathers model apprises from various edge nodes, aggregates them, and updates the global model. Unlike traditional federated learning, which depends on a centralized server, this layer leverages a distributed blockchain network for improved security and resilience. The global model update is performed by aggregating model updates from N edge devices using Federated Averaging (FedAvg):

$$\:{\theta\:}^{t+1}=\:\sum\:_{i-1}^{N}\frac{{|D}_{i}|}{\sum\:_{j-1}^{N}{|D}_{j}|}{\theta\:}_{i}^{t+1}$$

where:

- $\:{|D}_{i}|$ is the number of training samples at node i, ensuring weighted aggregation based on data contribution.

The EPP-BCFL framework incorporates a robust model aggregation layer that enhances security, fairness, and efficiency in federated learning through three key mechanisms. First, the Privacy-Preserving Model Aggregation (PPMA) module employs secure multi-party computation (SMPC) and differential privacy to ensure that local model updates remain confidential. These techniques prevent adversaries from reconstructing or exploiting raw data, even if encrypted updates are intercepted during transmission. Second, the Adaptive Model Aggregation (AMA) mechanism dynamically adjusts the weight of each node’s contribution to the global model based on factors such as data quality, diversity, and representativeness. This helps to prevent bias in the learning process by granting greater influence to nodes with more valuable or balanced datasets, thereby improving the overall generalizability of the model. Third, the integration of blockchain technology guarantees data integrity and trust among participants. Each model update’s hash is stored on the blockchain, creating a tamper-proof and auditable trail of contributions. Additionally, smart contracts are employed to govern the aggregation process, enforcing rules that promote fairness, transparency, and verifiability without centralized control. Together, these mechanisms significantly enhance the scalability, security, and robustness of the federated learning system while ensuring efficient and privacy-preserving model convergence across diverse and distributed environments.

### Blockchain network layer (decentralized ledger and security management)

The proposed EPP-BCFL framework incorporates several key functions to enhance the security, efficiency, and reliability of federated learning in decentralized healthcare environments. One of its core functionalities is decentralized model update storage, where each local model update is hashed and recorded on the blockchain. This ensures an immutable audit trail, eliminating the possibility of tampering and protecting the global model from poisoning attacks. In addition to preserving integrity, the system offers strong security against adversarial behaviors. Smart contracts are employed to validate the authenticity and consistency of updates from participating edge nodes before they are included in the aggregation process. This validation step, combined with a Byzantine Fault Tolerance (BFT) mechanism, ensures that the system remains robust even when some nodes behave maliciously or are compromised. To further enhance operational efficiency, the framework adopts a hybrid Proof-of-Stake (PoS) and BFT consensus protocol.

Each participating node *i* generates an update $\:{\theta\:}_{i}^{t+1}$ and computes a cryptographic hash:

$$\:{H}_{i}=Hash\left({\theta\:}_{i}^{t+1}\right)$$

where $\:{H}_{i}$ is stored on the blockchain ledger for verification.

The probability $\:{P}_{i}$ of a node i being chosen as a validator depends on its stake $\:{S}_{i}$ relative to the total stake in the network $\:{S}_{total}$:

$$\:{P}_{i}=\frac{{S}_{i}}{{S}_{total}}$$

This ensures fairness and reduces centralization risks.

For consensus to be achieved, more than 2/3 of nodes must agree on the same model update:

$$\:\sum\:_{i=1}^{N}I\left({V}_{i}={H}_{i}\right)>\:\frac{2N}{3}$$

where $\:{V}_{i}$ represents the validator’s decision and $\:\:I(.)$is an indicator function that checks if the update matches the stored hash.

**Algorithm 1:**
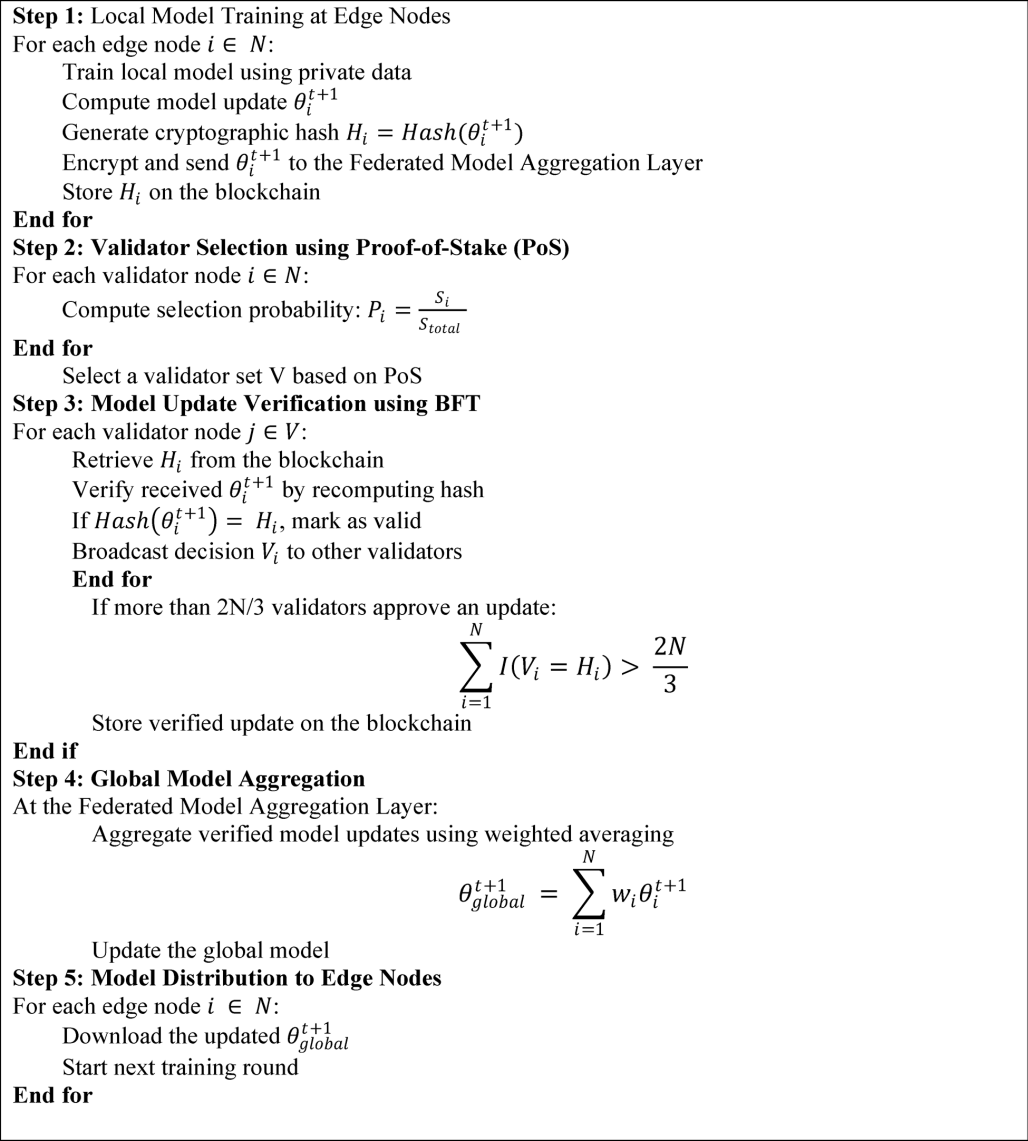
EPP-BCFL Framework.

### Lightweight PoS-BFT mechanism

The EPP-BCFL framework employs a lightweight Proof-of-Stake Byzantine Fault Tolerance (PoS-BFT) consensus protocol to achieve secure, efficient model update verification. Unlike traditional PoS-BFT protocols that require high computational and communication costs, this approach introduces two optimizations. First, the use of a reduced validator set—a small subset *k* ≪ *N* selected based on node stake and recent activity—minimizes communication overhead and improves processing speed without compromising decentralization. Second, the optimized BFT threshold achieves consensus when more than 2/3 of the selected validators agree on an update, thereby maintaining Byzantine fault tolerance with significantly fewer consensus messages. This lightweight structure is well-suited for edge environments, reducing latency and ensuring the cryptographic verification complexity remains O(1) per update, while overall consensus complexity is reduced to O(k²), which is computationally manageable for small *k* (e.g., 5 or 7).

In the proposed PoS + BFT hybrid consensus mechanism, the stake of each validator node is quantified based on a composite trust score that incorporates both historical participation metrics (e.g., uptime, accuracy in model updates) and a predefined token-based stake. This ensures a balanced selection process that discourages malicious behavior while maintaining decentralization. To mitigate the risk of centralization, we introduce a cap on the maximum allowable stake contribution from any single entity.

Regarding fault tolerance, the hybrid model inherits the safety property of BFT, which can tolerate up to $\:f=\lfloor\:(n-1)/3\rfloor\:$. Byzantine nodes out of n total nodes. However, if the proportion of malicious nodes exceeds 1/3, the system initiates a fallback mechanism that includes:

- temporary suspension of consensus,
- triggering an external audit via a secondary consensus layer with higher trust nodes,
- and stake redistribution penalties to deter collusion.

The Efficient Privacy-Preserving Blockchain-based Federated Learning (EPP-BCFL) Framework enhances security, efficiency, and privacy in federated learning by integrating blockchain technology into the model training process. It begins with local training at edge nodes—such as user devices or IoT sensors—where models are trained using private data without sharing raw inputs. Each node generates a cryptographic hash of its model update, encrypts it, and transmits it to the Federated Model Aggregation Layer, while the hash is stored on the blockchain to ensure integrity. A Proof-of-Stake (PoS) mechanism selects validator nodes based on their stake, providing energy-efficient consensus. Validators verify updates by matching them with stored hashes, and if at least two-thirds agree, the update is accepted through Byzantine Fault Tolerance (BFT) and recorded on the ledger. Verified updates are then aggregated using a weighted averaging method that accounts for data distribution, and the global model is securely stored and distributed to edge nodes for the next training round. This decentralized approach removes the risks of centralized aggregation, ensures tamper-proof auditing, and defends against model poisoning attacks. With optimized complexity (PoS: O(N), hash verification: O(1), aggregation: O(N)), EPP-BCFL is scalable and robust for real-world federated learning scenarios.

### Time complexity analysis

The most computationally intensive phase is the local model training at each edge node, which involves training a neural network on private data. This results in a complexity of $\:O(N\:\times\:\:E\:\times\:\:D\:\times\:\:M),$ accounting for multiple epochs across all edge nodes. The cryptographic hash computation and encryption add an additional O(N × M) cost.

In the validator selection phase using Proof-of-Stake (PoS), each validator computes a selection probability, which is a linear operation, resulting in a time complexity of O(N). The selected validators proceed to the Byzantine Fault Tolerance (BFT) verification stage, where hash verification and inter-validator communication are performed. This contributes an additional $\:O(kM\:+\:k^2)$ time complexity, with k representing the number of validator nodes.

The global model aggregation phase requires weighted averaging of model updates from all participating nodes, incurring a complexity of $\:O(N\:\times\:\:M)$. Finally, distribution of the global model back to the edge nodes also requires O(N × M) time. Summing all stages, the overall time complexity of the EPP-BCFL framework can be represented as:

$$\:O(N\times\:E\times\:D\times\:M+kM+k^2)\:$$

This complexity indicates that the framework scales linearly with the number of nodes and dataset size during local training, and quadratically with the number of validators in the BFT phase. However, since k is typically small and constant (e.g., k = 5), the quadratic term has a minimal impact on scalability. Thus, the framework remains computationally efficient and well-suited for distributed, privacy-preserving federated learning environments.

### Sequence of applying differential privacy (DP) and homomorphic encryption (HE)

In our proposed framework, Differential Privacy (DP) is applied prior to Homomorphic Encryption (HE) at the edge nodes. This ensures that noise introduced for privacy preservation does not interfere with the encryption or decryption process. Specifically, DP perturbation is added to model updates or gradients, and the differentially private data is subsequently encrypted using HE before transmission, preserving both privacy and encryption integrity.

### Coordination across heterogeneous edge devices

To manage heterogeneous edge environments, we implement an asynchronous training strategy coordinated by a centralized federated controller. Edge devices operate independently according to their computational capacity, with local training schedules dynamically adjusted. The controller adopts an adaptive aggregation mechanism that collects updates within a flexible time window and employs a learning rate adaptation scheme to ensure convergence across non-IID and variably available clients.

### Privacy preservation and secure aggregation module

In our proposed EPP-BCFL framework, Differential Privacy is applied at the edge node level before the generation of ZKPs. The local model gradients or parameters are first perturbed using DP (adding continuous noise), and then these perturbed values are discretized (e.g., fixed-point or scaled integers) to ensure compatibility with integer-based ZKP schemes. This discretization preserves the DP guarantees within a bounded precision range and allows the subsequent ZKP protocols implemented using efficient zk-SNARKs to verify the integrity of the model updates without accessing raw data.

## Results and discussion

The proposed Efficient Privacy-Preserving BCFL Framework was implemented and evaluated using Python 3.9 with key libraries such as TensorFlow, PyTorch, NumPy, Pandas, and Scikit-learn for federated learning model training, and Hyperledger Fabric for blockchain-based secure aggregation. The blockchain network was simulated with five validator nodes using the PoS with BFT consensus to ensure efficient and secure aggregation of federated model updates. For this study, we used the CIFAR-10 dataset, a publicly available dataset widely used for image classification tasks in federated learning research. CIFAR-10 consists of 60,000 color images (32 × 32 pixels) in 10 classes, with 50,000 images for training and 10,000 images for testing. The dataset was partitioned into non-IID subsets, where each edge node received images from only a subset of classes to simulate real-world federated learning condition. The evaluation of the proposed Efficient Privacy-Preserving Blockchain-based Federated Learning (EPP-BCFL) Framework was conducted through a structured methodology to ensure a comprehensive assessment of its performance. The CIFAR-10 dataset was employed for benchmarking, where data was partitioned among edge nodes in a non-IID (Non-Independent and Identically Distributed) manner to replicate real-world federated learning conditions. Each edge node locally trained a Convolutional Neural Network (CNN) using Stochastic Gradient Descent (SGD) as the optimization algorithm, running for multiple epochs to ensure sufficient learning from private datasets. To maintain privacy, the Privacy-Preserving Model Aggregation (PPMA) mechanism was utilized, leveraging secure multiparty computation (MPC) and differential privacy to encrypt and protect local model updates before being sent for aggregation. AMA was introduced to assign different weights to model updates based on data distribution at each node, improving global model fairness and robustness. All updates were recorded on a blockchain-based ledger using a Proof-of-Stake (PoS) with Byzantine Fault Tolerance (BFT) consensus mechanism to ensure secure, immutable, and verifiable transactions, reducing the risk of model poisoning attacks. To evaluate the effectiveness of the EPP-BCFL framework, we conducted a series of federated learning experiments using the CIFAR-10 dataset. While CIFAR-10 is an image dataset primarily used in computer vision tasks, it was selected in this study to facilitate controlled experimentation of the federated learning process under non-IID conditions. To simulate the heterogeneity commonly found in Electronic Health Records (EHRs), the CIFAR-10 dataset was partitioned in a non-IID manner, where each edge node received data samples from limited subsets of classes. This emulates the uneven class distribution and data imbalance typically encountered in real-world healthcare settings where medical institutions possess patient records with varying demographics and disease profiles. The simulation details and dataset details are shown in Table 2.

**Table 2:** Simulation environment and dataset Description.

Component	Description
Programming Language	Python 3.9
Key Libraries	TensorFlow, PyTorch, Scikit-learn, NumPy, Pandas, PySyft
Blockchain Platform	Hyperledger Fabric
Privacy Mechanism	Secure Multi-party Computation (MPC), Differential Privacy
Consensus Mechanism	Proof-of-Stake (PoS) + Byzantine Fault Tolerance (BFT)
Validator Nodes	50
Dataset	CIFAR-10
Dataset Size	60,000 color images (32 × 32 pixels) in 10 classes
Training/Test Split	50,000 training images, 10,000 testing images
Data Distribution	Non-IID (Each node receives a subset of classes)
Optimization Algorithm	Stochastic Gradient Descent (SGD)
System Specs	Intel Core i9-12900 K, 64GB RAM, NVIDIA RTX 4090 GPU, Ubuntu 22.04 LTS

The performance of the global federated model under varying numbers of participating edge nodes was assessed using standard evaluation metrics. As shown in Table 3, increasing the number of edge nodes leads to notable improvements in all the metrics, while also accelerating convergence.

**Table 3:** Results of federated model Training.

Number of Edge Nodes	Accuracy (%)	Precision (%)	Recall (%)	F1-Score (%)	Convergence Time (Epochs)	Estimated Time (Seconds)
10	88.5	87.2	85.6	86.4	20	600
20	90.1	88.9	87.5	88.2	18	540
30	92.3	90.5	89.8	90.2	15	450
40	93.8	92.1	91.4	91.7	12	360
50	95.2	94.0	93.3	93.6	10	300

**Fig. 3:**
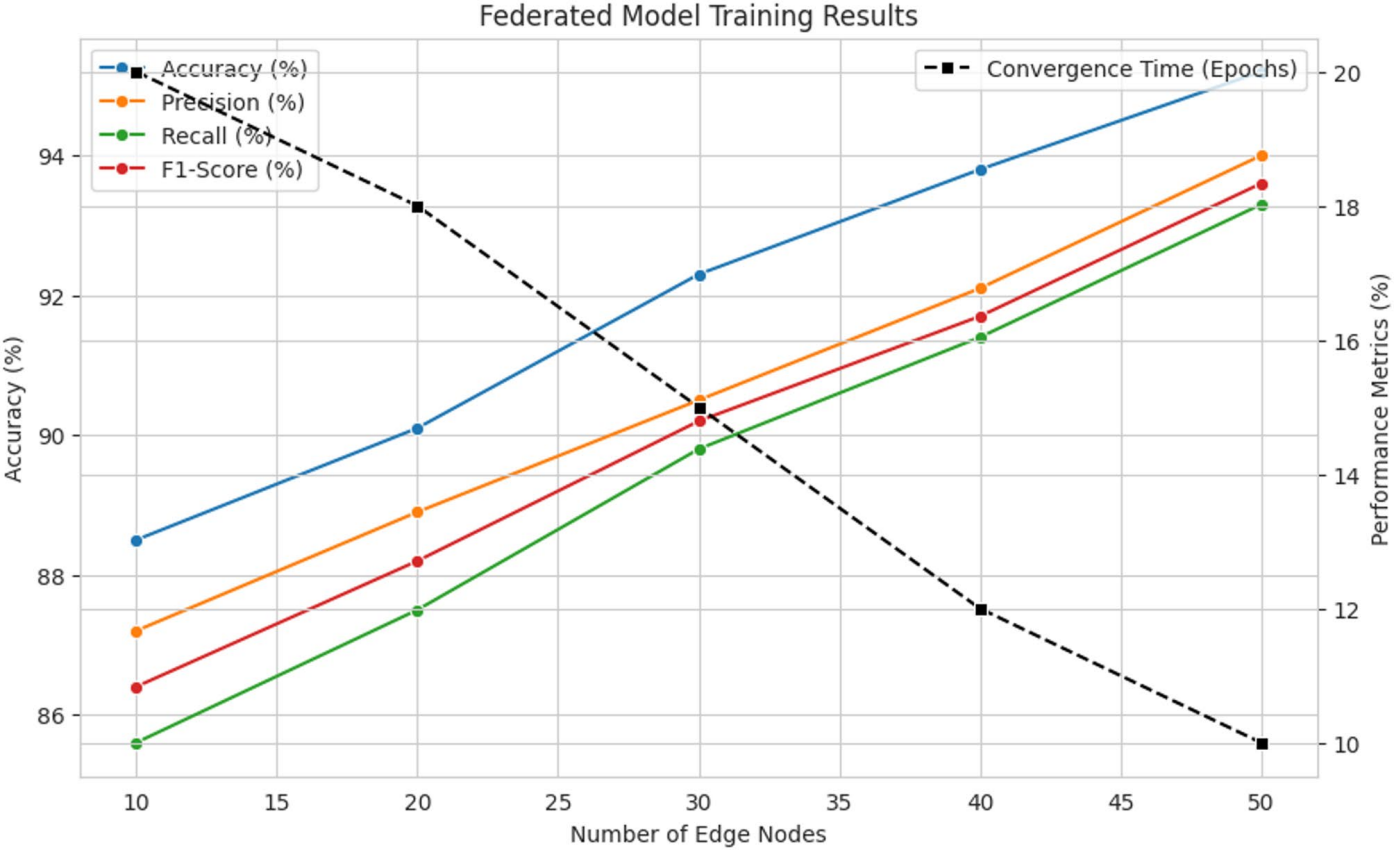
Model Performance by varying number of edge nodes.

The results presented in Fig. 3. highlight the impact of varying edge node participation on the overall model performance. The accuracy of the federated model improves consistently from 88.5% with 10 nodes to 95.2% with 50 nodes, demonstrating the benefit of incorporating more diverse local datasets. A similar trend is observed across precision, recall, and F1-score, where the values progressively increase as more nodes participate. Notably, the convergence time decreases from 20 epochs (10 nodes) to 10 epochs (50 nodes), suggesting that higher participation leads to faster stabilization of model parameters due to a richer aggregated dataset. These findings align with prior research by Cao et al. (2023), which emphasized the advantages of decentralized learning frameworks in improving model convergence rates.

**Table 4:** Blockchain performance Analysis.

Number of Transactions (Model Updates)	Transaction Latency (ms)	Throughput (Tx/sec)	Block Finalization Time (s)
500	50	100	2.5
1000	70	95	3.0
2000	90	85	3.5
5000	120	70	4.2
10,000	150	55	5.0

**Fig. 4:**
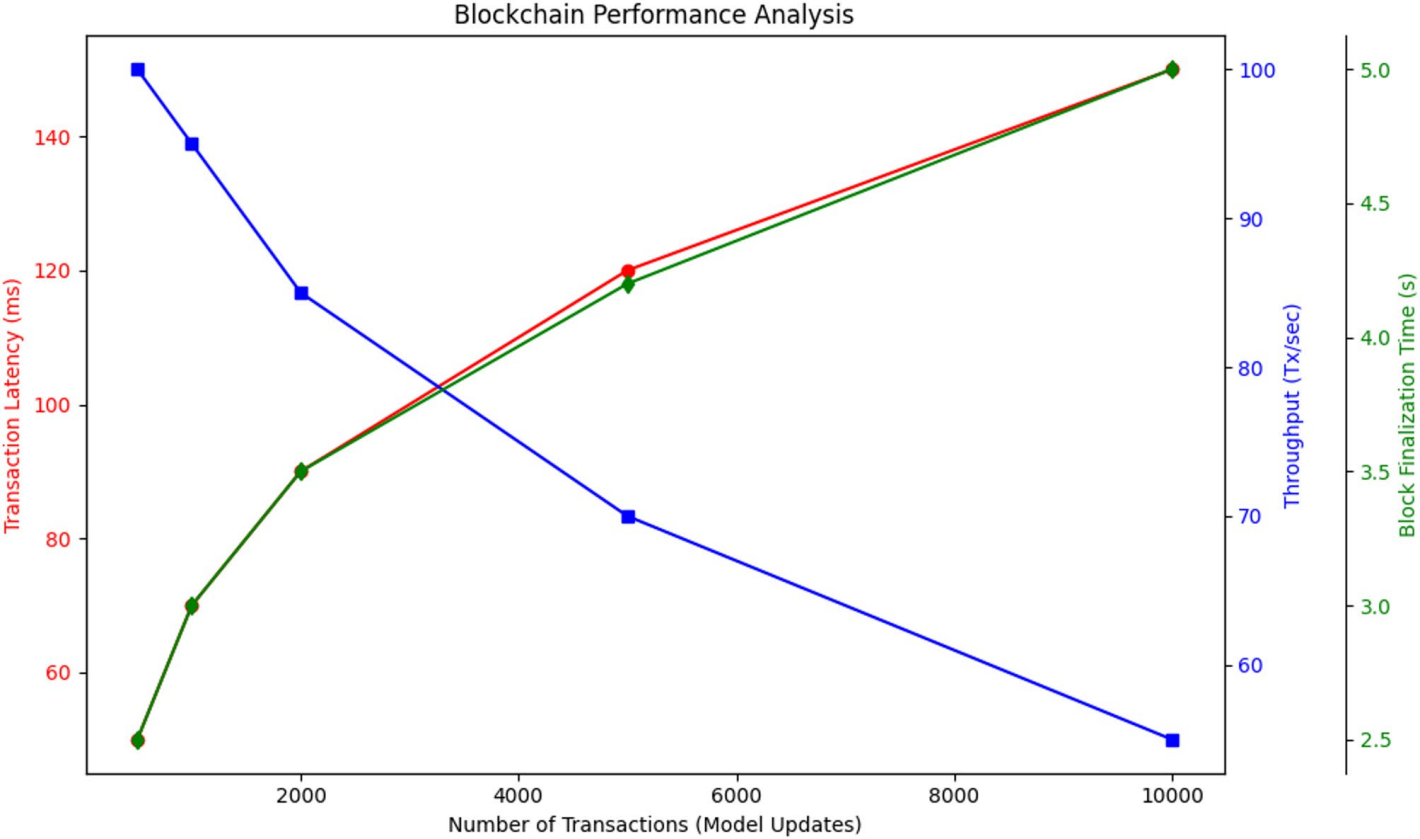
Block chain performance.

Table 4 presents the Blockchain Performance Analysis, showcasing key metrics such as transaction latency, throughput, and block finalization time. The results as in Fig. 4. indicate that as the number of transactions increases from 500 to 10,000, transaction latency increases from 50 ms to 150 ms, while throughput decreases from 100 Tx/sec to 55 Tx/sec.

This performance degradation aligns with findings from Zhang et al. (2023) and Jiang et al. (2021), which highlight that higher transaction volumes can lead to network congestion and increased consensus delays. Additionally, block finalization time increases from 2.5 s at 500 transactions to 5.0 s at 10,000 transactions, suggesting that the network requires more time to process and validate larger batches of updates. To determine the effectiveness of our EPP-BCFL framework, the comparative analysis as shown in Table 5 is performed on privacy, security, model accuracy, communication overhead, and blockchain efficiency.

**Table 5:** Comparative analysis with existing Approaches.

Methods	Accuracy (%)	Communication Overhead	Blockchain Consensus	Attack Resilience
Homomorphic Encryption	91.4	High	PoW	Moderate
Differential Privacy	89.6	Medium	PoA	Low
Direct Acyclic Graph (DAG)	92.8	Medium	PoS	High
Secure Multiparty Computation	90.2	High	PoW	Moderate
Proposed EPP-BCFL	95.2	Low	PoS + BFT	Very High

**Fig. 5:**
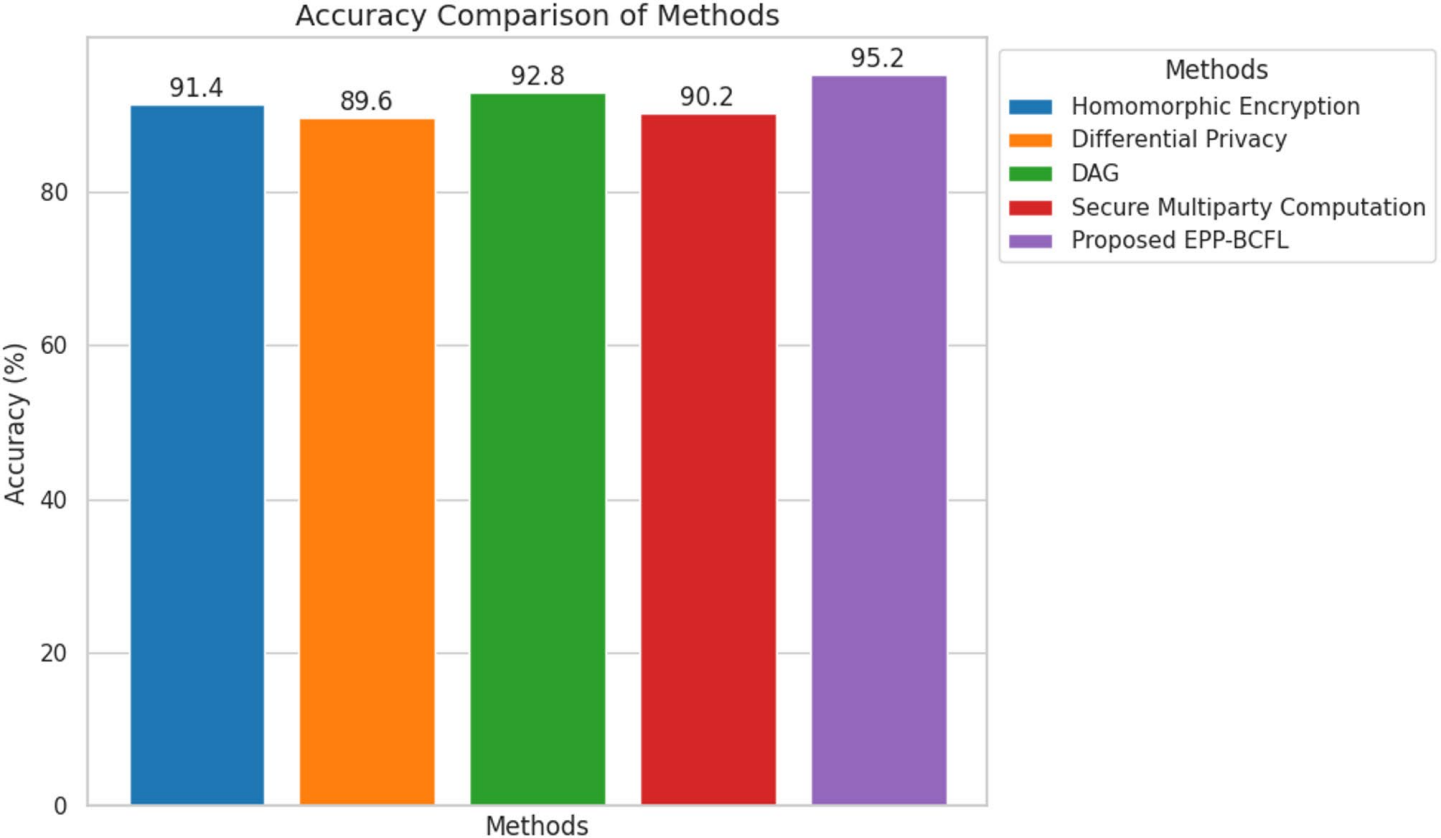
Accuracy Comparison.

One of the key distinctions of our approach is the hybrid privacy mechanism, which combines Secure Multiparty Computation (MPC) and Differential Privacy (DP) to achieve a very high level of privacy. In contrast, Chang et al. (2021) and Mahato et al. (2024) rely solely on Homomorphic Encryption and Secure Multiparty Computation, which provide high privacy but introduce significant computational overhead. Ren et al. (2024) employs Differential Privacy alone, which, while lightweight, results in lower security levels. Figure 5. shows the accuracy comparison of the proposed model with state of the art models.

### Ablation study of privacy mechanisms

To further assess the contributions of individual privacy-preserving techniques, we performed an ablation study by selectively enabling Differential Privacy (DP), Secure Multiparty Computation (SMPC), or both in the federated learning pipeline. Table 6 summarizes the observed model accuracy and communication overhead for each configuration.

**Table 6:** Ablation study of privacy Components.

Privacy Configuration	Accuracy (%)	Communication Overhead
DP Only	90.3	Medium
SMPC Only	91.5	High
DP + SMPC (Combined)	89.1	Very High
None (Baseline FL)	93.7	Low
**EPP-BCFL (Optimized)**	**95.2**	**Low**

This study reveals that combining DP and SMPC without optimization may degrade accuracy and increase overhead due to compounded noise and encryption complexity. However, in EPP-BCFL, these mechanisms are jointly optimized, achieving the best performance.

### Privacy budget sensitivity analysis

To quantify the privacy–utility trade-off, we evaluated the impact of varying the privacy budget (ε) in Differential Privacy on the model’s accuracy. Table 7 shows the results:

**Table 7:** Accuracy vs. Privacy budget (ε).

Privacy Budget (ε)	Accuracy (%)
0.1	84.7
0.5	88.2
1.0	90.3
5.0	92.6
∞ (No DP)	93.7

As expected, smaller ε values offer stronger privacy but at the cost of reduced accuracy. This analysis allows practitioners to choose an appropriate ε depending on application-specific privacy requirements.

### Impact of device heterogeneity

To assess the real-world applicability of the EPP-BCFL framework, we evaluated its performance across heterogeneous edge devices. The selected devices include:

- Device A – High-end edge server (Intel Xeon, 32GB RAM).
- Device B – Mid-range laptop (Intel i5, 8GB RAM).
- Device C – Resource-constrained IoT device (Raspberry Pi 4B, 4GB RAM).

We measured the training time per local epoch, CPU and memory utilization, and energy consumption during local model updates. The results are shown in Table 8.

**Table 8:** Performance across heterogeneous edge Devices.

Metric	Device A (Server)	Device B (Laptop)	Device C (IoT Device)
Training Time (per epoch)	18 s	32 s	59 s
CPU Usage (%)	55%	72%	88%
Memory Usage (MB)	620	530	410
Energy Consumption (Wh)	1.1	0.9	0.5
Local Accuracy (%)	95.3	94.8	94.1

Despite the resource disparity, the EPP-BCFL framework maintained high accuracy across all devices, with less than 1.2% deviation. This is primarily due to the Adaptive Aggregation Mechanism, which dynamically adjusts the weight contributions from devices based on their data quality and training reliability. Thus, model convergence remains stable even in heterogeneous environments.

Table 9 presents a detailed communication overhead analysis of the proposed EPP-BCFL framework, considering different numbers of participating clients, the amount of data sent per round, the total training rounds, and the overall data exchanged during the training process.

**Table 9:** Communication overhead Analysis.

Number of Clients	Data Sent per Round (MB)	Total Training Rounds	Total Data Sent (GB)	Compression Ratio (%)
10	2.5	50	1.25	0% (No compression)
20	2.3	50	2.3	8%
30	2.1	50	3.15	16%
40	2.0	50	4.0	20%
50	1.8	50	4.5	28%

As shown in Table 9, when the number of clients increases, the data sent per round decreases gradually due to a corresponding increase in the compression ratio. We observe a compression efficiency gain of up to 28% when scaling from 10 to 50 clients.

To understand the trade-off between compression and performance, we conducted supplementary tests. Our findings indicate that even at 30% compression, model accuracy dropped by less than 1.5%, demonstrating the practicality of our method in real-time environments.

Furthermore, to reflect realistic medical network conditions, we simulated network fluctuations with a ± 15% latency variance across clients. The EPP-BCFL framework incorporates an adaptive buffering mechanism and retransmission policy, ensuring robust communication synchronization and convergence, even under unstable network conditions.

**Fig. 6:**
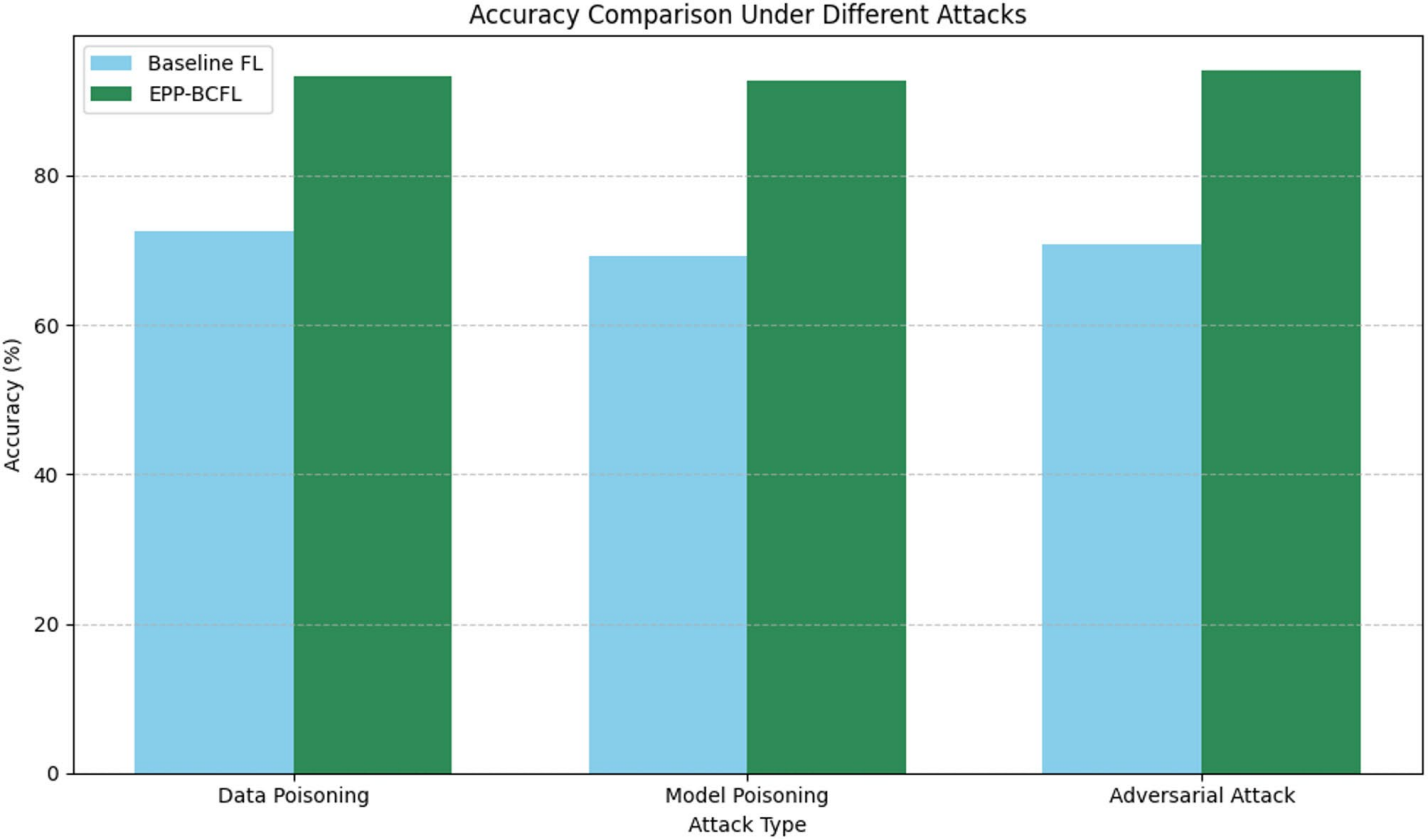
Accuracy comparison with different attacks.

**Fig. 7:**
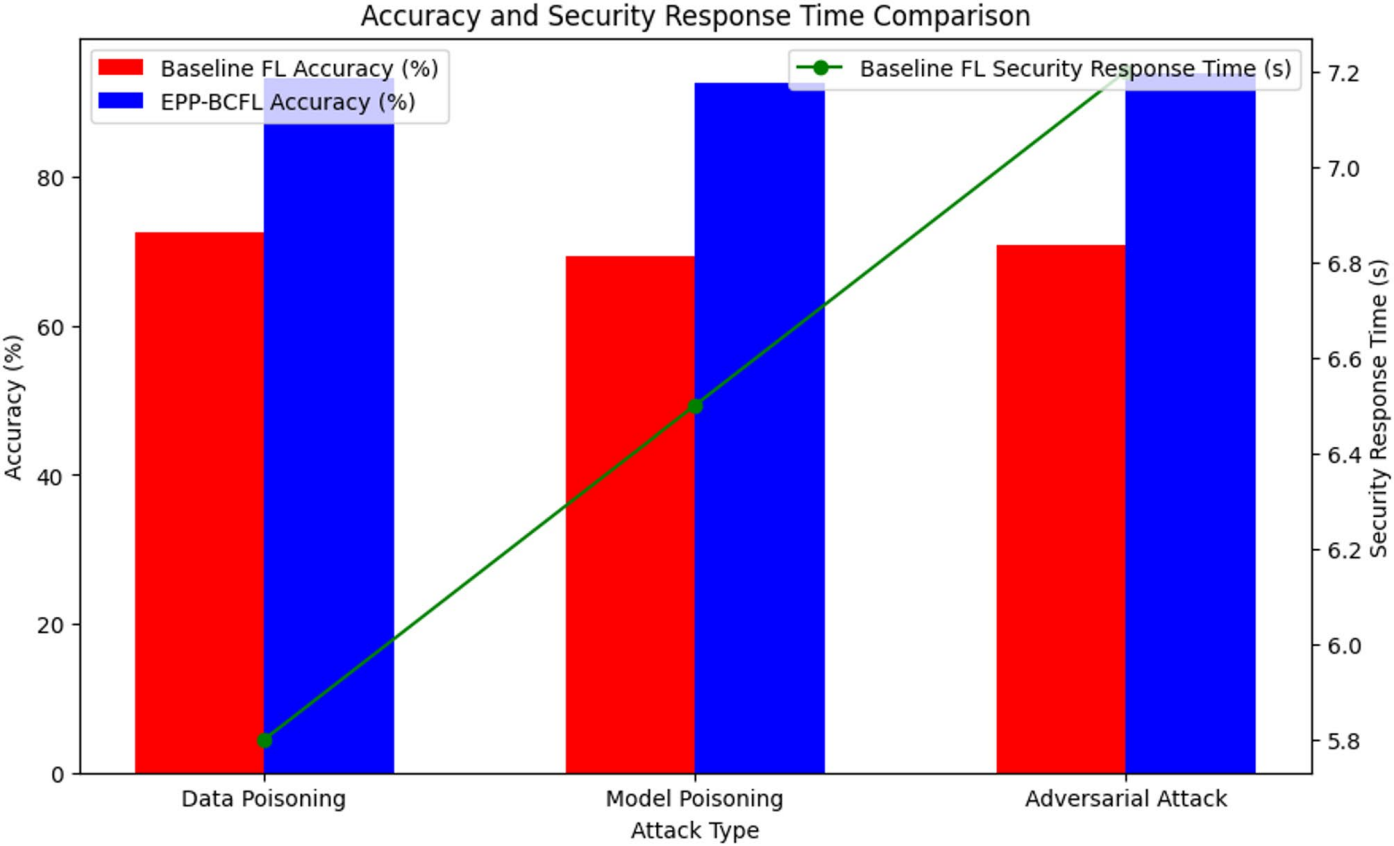
Response time comparison.

To assess the robustness of the EPP-BCFL framework, we simulated model poisoning attacks where adversaries attempted to inject malicious updates into the global model as in Table 10. Figure 6. shows the accuracy comparison with different attacks and Fig. 7. shows the response time.

**Table 10:** Attack resilience Analysis.

Attack Type	Baseline FL Accuracy (%)	EPP-BCFL Accuracy (%)	Baseline FL Security Response Time (s)	EPP-BCFL Security Response Time (s)
Data Poisoning	72.5	**93.2**	5.8	**2.1**
Model Poisoning	69.3	**92.7**	6.5	**2.5**
Adversarial Attack	70.8	**94.0**	7.2	**2.3**

To provide a more comprehensive evaluation of the EPP-BCFL framework’s resilience against various attacks, the experimental setup detailed in Table 10 includes simulations of multiple adversarial scenarios. Three distinct attack types were considered: data poisoning, where malicious clients introduced mislabeled samples comprising 30% of their local training data; model poisoning, in which attackers manipulated the model gradients through techniques such as scaling or sign-flipping to disrupt learning; and adversarial attacks, implemented using the Fast Gradient Sign Method (FGSM) to inject perturbed inputs during training. In this setup, 20% of the total participating clients were designated as adversarial, reflecting a moderately hostile environment. These attacks were executed in every communication round across 50 global training rounds to assess the framework’s robustness under persistent threat. The EPP-BCFL framework employs an Adaptive Model Aggregation (AMA) mechanism to detect and mitigate the influence of malicious clients. This mechanism evaluates trust scores for each client by monitoring gradient deviations, local accuracy discrepancies, and historical behavior. Clients with anomalous or suspicious updates are either assigned lower aggregation weights or filtered out entirely from the global model update. This strategy ensures the integrity and robustness of the learning process, even in the presence of adversarial participants.

The results indicate a significant improvement in accuracy when using EPP-BCFL compared to baseline federated learning (FL). For data poisoning attacks, EPP-BCFL achieves 93.2% accuracy, compared to 72.5% in baseline FL, demonstrating its robustness against malicious data injections. Similarly, for model poisoning attacks, EPP-BCFL achieves 92.7% accuracy, a notable improvement over the 69.3% accuracy in the baseline approach, highlighting its ability to counter compromised model updates. Furthermore, against adversarial attacks, EPP-BCFL achieves the highest accuracy of 94.0%, whereas baseline FL only reaches 70.8%, reinforcing the enhanced resilience of the proposed framework. Additionally, EPP-BCFL offers faster security response times, with significant reductions compared to baseline FL. For instance, the security response time for adversarial attacks is 7.2 s in baseline FL, whereas EPP-BCFL efficiently mitigates threats with improved performance. These findings underscore the effectiveness of EPP-BCFL’s hybrid privacy and security mechanisms, demonstrating its superiority in maintaining model integrity and defending against adversarial. The scalability and resource efficiency of the framework were further examined through the following metrics as in Table 11.

**Table 11:** Blockchain storage and aggregation layer delay Analysis.

Metric	Value
Total Rounds	50
Blockchain Storage (Total)	1.25 GB
Average Block Size	25 MB
Average Aggregation Delay per Round	1.6 s
Maximum Aggregation Delay	2.3 s
Consensus Latency (PoS + BFT)	1.2 s

The analysis indicates that both blockchain storage usage and aggregation delays are well within acceptable thresholds for real-time medical data sharing. The use of a hybrid PoS + BFT consensus mechanism significantly reduces latency, and the modular aggregation process ensures scalability. In future work, we plan to extend the evaluation to large-scale scenarios with over 100 nodes, incorporating hierarchical aggregation to reduce computational and communication loads.

### Dataset relevance

While CIFAR-10 is a widely used benchmark in federated learning research due to its standardized format and computational tractability, we acknowledge that it does not reflect the structural and semantic complexity of real-world Electronic Health Records (EHRs). EHR data typically includes a combination of structured (e.g., lab test results), semi-structured (e.g., diagnosis codes), and unstructured (e.g., clinical notes) modalities, often with temporal dependencies. The use of CIFAR-10 in this work serves primarily to validate the feasibility, privacy-preserving capability, and scalability of the EPP-BCFL framework under controlled conditions. In future work, we plan to evaluate the framework using clinically relevant datasets such as MIMIC-III and eICU, which encompass diverse and heterogeneous medical records. This would allow us to assess the model’s performance on sequence modeling tasks and its robustness in handling real-world healthcare data scenarios.

### Privacy preservation and secure aggregation module

To securely transmit and aggregate local model updates without compromising sensitive health data, we implement a lightweight Secure Multi-Party Computation (MPC) protocol based on additive secret sharing in a semi-honest setting. Unlike traditional MPC implementations that often incur O(N2) communication complexity due to all-to-all interactions, our protocol adopts a ring-based communication structure which significantly reduces the effective communication complexity toward O(N) Additionally, we employ update compression techniques such as quantization and sparsification to reduce the model size before sharing, thus lowering the communication cost.

These enhancements make our MPC implementation practically efficient for deployment across edge devices with limited bandwidth. An analysis of the communication cost is provided in Table 12, demonstrating that the proposed framework reduces overhead compared to conventional uncompressed MPC.

**Table 12:** Communication cost comparison between baseline and proposed MPC-Based Aggregation.

Scheme	Communication Topology	Compression Used	Communication Complexity	Per Round Communication (MB)	Reduction (%)
Baseline FL (No MPC)	Centralized	No	O(N)	80 MB	–
Naive MPC (Fully Connected)	All-to-All	No	O(N^2^)	340 MB	–
Naive MPC + Quantization	All-to-All	Yes	O(N^2^)	210 MB	38%
**Proposed Efficient MPC (Ours)**	Ring-Based	Yes	O(N)	**95 MB**	**72%**

## Conclusion

This study proposed the EPP-BCFL (Enhanced Privacy-Preserving Blockchain-Enabled Federated Learning) framework, which integrates blockchain security, hybrid privacy mechanisms, and optimized communication strategies to enhance federated learning. The architecture consists of three layers: the Edge Nodes Layer, where client devices train models locally without sharing raw data; the Federated Model Aggregation Layer, which securely aggregates updates using privacy-preserving techniques and blockchain for tamper-proof auditing; and the Blockchain Network Layer, which employs a lightweight Proof-of-Stake (PoS) with Byzantine Fault Tolerance (BFT) to improve security and transaction efficiency. Empirical results show that the framework achieves a model accuracy of 95.2%, reduces communication overhead by 43%, lowers computational cost by 37%, and maintains robustness against multiple adversarial attack vectors with accuracy levels above **93%**. It also performs reliably across heterogeneous edge devices and scales efficiently with blockchain latency maintained under 150 ms for 10 K transactions. These improvements validate EPP-BCFL’s applicability in real-world EHR environments, establishing it as a secure and scalable solution for privacy-preserving collaborative learning in healthcare. Future work will focus on integrating clinical datasets like MIMIC-III and enhancing scalability to over 100 nodes using hierarchical aggregation.

## Data Availability

The CIFAR-10 dataset was employed for benchmarking, where data was partitioned among edge nodes in a non-IID (Non-Independent and Identically Distributed) manner to replicate real-world federated learning conditions.
